# Advancing driver fatigue detection in diverse lighting conditions for assisted driving vehicles with enhanced facial recognition technologies

**DOI:** 10.1371/journal.pone.0304669

**Published:** 2024-07-10

**Authors:** Ning Lin, Yue Zuo

**Affiliations:** Nanning University, Nanning, Guangxi, China; University of New South Wales, AUSTRALIA

## Abstract

Against the backdrop of increasingly mature intelligent driving assistance systems, effective monitoring of driver alertness during long-distance driving becomes especially crucial. This study introduces a novel method for driver fatigue detection aimed at enhancing the safety and reliability of intelligent driving assistance systems. The core of this method lies in the integration of advanced facial recognition technology using deep convolutional neural networks (CNN), particularly suited for varying lighting conditions in real-world scenarios, significantly improving the robustness of fatigue detection. Innovatively, the method incorporates emotion state analysis, providing a multi-dimensional perspective for assessing driver fatigue. It adeptly identifies subtle signs of fatigue in rapidly changing lighting and other complex environmental conditions, thereby strengthening traditional facial recognition techniques. Validation on two independent experimental datasets, specifically the Yawn and YawDDR datasets, reveals that our proposed method achieves a higher detection accuracy, with an impressive 95.3% on the YawDDR dataset, compared to 90.1% without the implementation of Algorithm 2. Additionally, our analysis highlights the method’s adaptability to varying brightness levels, improving detection accuracy by up to 0.05% in optimal lighting conditions. Such results underscore the effectiveness of our advanced data preprocessing and dynamic brightness adaptation techniques in enhancing the accuracy and computational efficiency of fatigue detection systems. These achievements not only showcase the potential application of advanced facial recognition technology combined with emotional analysis in autonomous driving systems but also pave new avenues for enhancing road safety and driver welfare.

## 1 Introduction

### 1.1 Background

In the advancement of intelligent driving assistance systems, monitoring driver fatigue has emerged as a crucial technological and ethical challenge to ensure road safety. This technology’s evolution not only safeguards driving safety but also profoundly embodies the respect for human life and dignity. Through ongoing scientific research, our comprehension of fatigue driving’s intricate nature has significantly expanded, particularly under rapidly changing lighting conditions, such as when drivers encounter varying levels of brightness (e.g., entering tunnels or when surrounded by diverse structures like buildings and overpasses). In these contexts, the yawning behavior of drivers, a key indicator of fatigue, introduces an augmented risk factor. Such sudden lighting changes can impair a driver’s visual adaptability and focus, thus elevating the risk of accidents. This scenario underscores the critical role of fatigue monitoring technology in promoting driving safety and highlights the challenges in effective monitoring within specific environmental settings.

Firstly, a study by Azam et al. (2014) highlighted that fatigue-related traffic accidents account for 10% on regular roads and 28% on highways, underscoring the varying impact of driver fatigue across different road types and setting the stage for targeted fatigue monitoring under diverse lighting conditions [1]. Subsequently, Liu et al. (2016) introduced a real-time fatigue detection method utilizing extreme learning machines, marking a significant technological advance and providing a crucial reference for adapting fatigue monitoring to changes in lighting [2]. The significance of in-vehicle warning systems, especially in scenarios marked by significant lighting shifts, was further underlined by Richardson (2019) [3]. Research by Vasile Plămădeală et al. [4] presented that 75% of fatal accidents are attributed to human factors such as fatigue, particularly in conditions of night driving or sudden lighting changes, thereby significantly heightening the risk of fatigue-induced accidents. Lastly, Davidović et al. (2020) found that fatigue driving contributes to 26% of all traffic accidents, reinforcing the critical need for fatigue monitoring technologies to bolster road safety amidst variable lighting environments [5].

This body of work not merely aggregates data and developments but serves as a crucial reminder of the collective responsibility shared by drivers towards ensuring the safety of all road users. It stresses the importance of leveraging technological advancements in fatigue detection to foster safer driving environments under diverse and challenging conditions.

### 1.2 Literature review

In the field of computer vision technology for driver fatigue detection, researchers have proposed various innovative methods in recent years (Kim et al. (2021) and Poulose et al. (2021)) [6, 7]. Zhang et al. (2015) used a fast and robust facial detection algorithm and Boost-LBP features for driver fatigue facial expression recognition, achieving significant results [8]. However, their method lacked robustness under complex lighting conditions, limiting its practical application range. Tao et al. (2017) introduced a method that aligned and normalized facial sequences to extract features related to fatigue expressions and used a sliding window for fatigue detection [9]. Despite improvements in feature extraction, this method’s computational efficiency in processing real-time video data still needed enhancement. Jia et al. (2021) proposed a fatigue detection method based on CNN-HMM, detecting the driver’s eyes, mouth, and head posture with an accuracy of 97.5% [10]. However, this method did not fully consider the impact of the driver’s emotional state on fatigue detection, an important dimension of fatigue detection.

Sacco et al. (2012) demonstrated a real-time non-invasive fatigue monitoring system using facial expressions, achieving an accuracy of 95.2% [11]. However, this system had limited adaptability to facial occlusions and varied expressions, potentially limiting its effectiveness under actual road conditions. Khan et al. (2014) proposed a comprehensive vision-based method to detect driver fatigue, achieving an average accuracy of 97.7% [12]. However, the adaptability of this method in complex environments, particularly under changing lighting and weather conditions, had not been fully verified. Qunzhu et al. (2019) developed an improved random forest cascade regression algorithm for detecting facial feature points in driver fatigue detection [13]. Although the method performed well in feature point detection, there was room for improvement in recognizing complex facial expressions and subtle fatigue signals. You et al. (2020) described a real-time driver fatigue detection algorithm based on facial motion entropy, achieving an accuracy of 94.32% [14]. Despite its high accuracy, the method’s computational efficiency and resource consumption in processing large volumes of real-time video data remained a challenge. Dong et al. (2021) proposed a method to detect driver fatigue and distraction, improving accuracy and computation time [15]. However, the universality and scalability of this method across different drivers and vehicle types had not been fully validated. Dong et al. (2022) discussed a method using random forests and convolutional neural networks to detect driver fatigue and distraction behaviors [16]. This research excelled in handling complex behavior analysis but still needed further study for real-time processing and low resource consumption.

Although existing research has made significant technological advancements, there are still deficiencies in handling facial recognition and emotion analysis in complex driving environments under changing lighting conditions (as shown in Table 1). In response to this challenge, we propose a comprehensive fatigue detection method combining advanced facial recognition technology and emotion state analysis, with a particular emphasis on accuracy and adaptability under drastic changes in lighting conditions.

**Table 1:** Summary of previous works and identified gaps.

Reference	Methodology	Identified Gaps
Zhang et al. (2015) [8]	Fast facial detection using Boost-LBP	Limited robustness under complex lighting conditions
Tao et al. (2017) [9].	Facial sequence alignment and normalization; sliding window for feature extraction	Needs improvement in computational efficiency for real-time data processing
Jia et al. (2021) [10]	CNN-HMM based detection focusing on eyes, mouth, and head posture	Lack of consideration for the driver’s emotional state
Sacco et al. (2012) [11]	Real-time monitoring using facial expressions	Limited adaptability to facial occlusions and varied expressions
han et al. (2014) [12]	Comprehensive vision-based method	Not fully verified adaptability in complex environments
Qunzhu et al. (2019) [13]	Improved random forest cascade regression for facial feature points detection	Improvement needed in recognizing complex facial expressions and subtle fatigue signals
You et al. (2020) [14]	Real-time detection based on facial motion entropy	Challenges in computational efficiency and resource consumption
Dong et al. (2021) [15]; Dong et al. (2022) [16].	Detection using random forests and CNNs	Need for real-time processing studies and verification of universality and scalability

### 1.3 Our contribution

This study is committed to breaking through the limitations of existing technologies in the field of driver fatigue monitoring, with a special focus on fatigue detection under changing lighting conditions. Our research contributions are mainly reflected in the following aspects:

1. Development of an advanced data preprocessing method based on deep learning technology, capable of extracting facial features with unprecedented accuracy under changing lighting conditions. This method not only enhances the accuracy of feature extraction but also strengthens the system’s stability under different lighting intensities and partial facial occlusions.
2. Introduction of an innovative dynamic keyframe extraction algorithm. Unlike traditional methods, our algorithm intelligently selects keyframes based on the dynamic changes in video content under changing lighting conditions. This approach significantly improves the efficiency of video data processing under varying lighting conditions, reducing the demand for computational resources and enabling real-time or near-real-time fatigue detection.
3. Design of an innovative composite action recognition network, combining multiple neural network technologies such as 3D convolutional networks and long short-term memory networks (LSTM), to enhance the recognition of subtle facial movements in continuous video frames under different lighting conditions. This network not only captures dynamic changes over time but also processes long-term temporal dependencies, significantly improving the accuracy of detecting minor facial movements related to fatigue driving under changing lighting conditions.

This paper is structured as follows: Section 1 introduces the study’s background and reviews relevant literature, identifying gaps our work aims to fill. In Section 2, we detail our proposed methodology for detecting driver fatigue, including data preprocessing, dynamic keyframe extraction, and the composite action recognition network. Section 3 shows the Algorithm Pseudocode. Section 4 presents experimental setups, datasets, and results, demonstrating the effectiveness of our approach. Finally, Section 5 concludes the paper, summarizing our findings and suggesting avenues for future research.

## 2 Our approach

### 2.1 Problem description

**Problem 1**. *In intelligent driving assistance systems, accurately identifying driver fatigue from facial behaviors (such as yawning, blinking, etc.) is challenging. Facial behaviors are critical indicators of fatigue states as they are usually involuntary and can be effectively captured by automated systems. We define the detection of driver fatigue states as a time series analysis problem, where each time point’s facial state can be represented by a multidimensional feature vector X*_*t*_. *Our goal is to design an algorithm that can accurately recognize fatigue patterns within these feature vectors*.

*Specifically, we need to solve the following mathematical problem*:

$$\begin{array}{c} F\left(X_{t}\right)=\sum_{i=1}^{N}w_{i}\cdot g\left(X_{t-i},\theta \right)+b \end{array}$$ (1)

*where F*(*X*_*t*_) *represents the fatigue detection function at time t*, *N is the size of the considered time window, w*_*i*_ *are weight parameters, g is a nonlinear function (e.g., convolutional neural network or recurrent neural network) used to extract relevant information from the facial feature vector X*_*t*−*i*_, *θ represents the parameters of function g, and b is a bias term*.

*We also need to consider the impact of environmental factors on fatigue detection, which can be expressed as*:

$$\begin{array}{c} E_{t}=\alpha \cdot L_{t}+\beta \cdot C_{t} \end{array}$$ (2)

*where E*_*t*_ *represents environmental factors at time t*, *L*_*t*_ *is the light intensity*, *C*_*t*_ *is the noise level inside the vehicle, and α and β are influence coefficients*.

*Therefore, the final fatigue detection model can be expressed as*:

$$\begin{array}{c} Y_{t}=\sigma \left(F\left(X_{t}\right)+E_{t}\right) \end{array}$$ (3)

*where Y*_*t*_ *is the fatigue state output at time t, and σ is an activation function (such as Sigmoid or Softmax) used to convert the model output into a probability of fatigue state*.

*Our goal is to minimize the prediction error, that is*:

$$\begin{array}{c} \text{min}\;\sum_{t=1}^{T}{\left(Y_{t}-{\hat{Y}}_{t}\right)}^{2} \end{array}$$ (4)

*where* ${\hat{Y}}_{t}$ *is the true fatigue state at time t, and T is the total observation time*.

### 2.2 Enhanced data preprocessing

#### 2.2.1 Motivation for data preprocessing enhancement

The advancement in driver fatigue detection is challenged by the complexity of driving environments and the diversity of driver behaviors, necessitating robust data preprocessing methods [17–19]. Traditional facial behavior recognition techniques often falter with complex, blurred, or dynamically changing expressions, influenced by varying lighting and camera angles. To address these issues, we propose an enhanced data preprocessing approach that leverages deep learning-based CNN technology for precise facial feature extraction and introduces emotion state analysis. This dual strategy allows for a more accurate and comprehensive detection of fatigue states, even in environments lacking direct environmental sensing, thereby significantly improving the reliability and robustness of fatigue detection systems.

#### 2.2 2. Mathematical principle

Under the enhanced data preprocessing framework, we utilize deep learning models to extract facial features and combine them with emotion state analysis to enhance the accuracy of fatigue detection. Deep learning models are chosen for their excellent performance in handling high-dimensional data and capturing complex patterns. This process can be described by the following mathematical models:

In our approach, the extraction of facial feature vectors and the analysis of emotion states are pivotal for enhancing the accuracy and reliability of fatigue detection. Instead of detailing these processes through complex mathematical equations, we simplify our explanation to make our methodology accessible to a wider audience.

The generation of the facial feature vector *V*_*t*_ is accomplished through the use of a CNN model. This process involves inputting a facial image *I*_*t*_ into the CNN, which is configured with a specific set of parameters (Φ). The CNN employs layers of neurons equipped with weights and biases to apply activation functions, such as the ReLU (Rectified Linear Unit), thereby extracting meaningful features from the facial image that are indicative of fatigue.

Following the feature extraction step, we analyze the emotional state of the driver using a Deep Neural Network (DNN), characterized by its parameters (Ψ). This network processes the extracted facial features *V*_*t*_ and utilizes functions like tanh (hyperbolic tangent) to interpret these features in the context of emotional states, contributing further to our understanding of the driver’s fatigue level.

These two models together constitute our enhanced data preprocessing framework, aiming to improve the accuracy and reliability of fatigue detection through in-depth analysis of facial features and emotion states.

Considering facial features and emotion states together, the fatigue detection problem can be redefined, and a theorem on the accuracy of fatigue detection and a corollary on the importance of data preprocessing can be proposed.

**Problem 2**. *Given a series of time-series facial image frames, we need to design an algorithm to accurately identify the driver’s fatigue state. This problem can be described as optimizing the following mathematical model*:

$$\begin{array}{cc} F^{\prime }\left(V_{t}\right) & =\;\sum_{i=1}^{M}u_{i}\cdot h\left(V_{t-i},\chi \right)+c\\ & =\;\sum_{i=1}^{M}u_{i}(\sum_{j=1}^{n}\alpha_{j}\cdot tanh\left(\beta_{j}\cdot V_{t-i}+\gamma_{j}\right))+c \end{array}$$ (5)

*With the integration of emotion state analysis, the fatigue detection model is updated as*:

$$\begin{array}{cc} Y_{t}^{\prime } & =\;\sigma (F^{\prime }\left(V_{t}\right)+E_{t})\\ & =\;\sigma (\sum_{i=1}^{M}u_{i}(\sum_{j=1}^{n}\alpha_{j}\cdot tanh\left(\beta_{j}\cdot V_{t-i}+\gamma_{j}\right))+c+E_{t}) \end{array}$$ (6)

*The goal is to minimize the prediction error*:

$$\begin{array}{c} \text{min}\;\sum_{t=1}^{T}{\left(Y_{t}^{\prime }-{\hat{Y}}_{t}\right)}^{2}=\;\text{min}\;\sum_{t=1}^{T}{\left(\sigma (\sum_{i=1}^{M}u_{i}(\sum_{j=1}^{n}\alpha_{j}\cdot tanh\left(\beta_{j}\cdot V_{t-i}+\gamma_{j}\right))+c+E_{t})-{\hat{Y}}_{t}\right)}^{2} \end{array}$$ (7)

**Theorem 1** (Accuracy of Fatigue Detection). *For any given continuous video sequence, if there is a time window size N and a sufficiently complex feature extraction network, it is possible to accurately identify the driver’s fatigue state through this network. In particular, if the feature extraction network can maximize the joint information entropy* *H*(*V*_*t*_, *E*_*t*_) *between the facial feature vector V*_*t*_ *and the emotion state E*_*t*_, *the identification of fatigue states will be more accurate*.

Proof is provided in the S1 Appendix.

**Corollary 1** (Importance of Data Preprocessing). *Let* M *represent the model in the fatigue detection system*, D *represent the original facial behavior dataset, and* P *represent the data preprocessing function. Then, effective data preprocessing* P *can significantly enhance the performance of* M *on the dataset* D.

Proof is provided in the S1 Appendix.

### 2.3 Dynamic keyframe extraction

#### 2.3.1 Motivation for dynamic keyframe extraction

Traditional facial behavior analysis and fatigue detection in rapidly changing environments pose significant challenges, particularly in processing high-frequency video data. The limitations of conventional methods become evident as they struggle with transient facial expressions and environmental lighting variations, often missing critical data [20, 21]. This necessitates a more dynamic approach to data processing, where keyframe extraction doesn’t just depend on static intervals but dynamically adapts to the content changes, especially in lighting. Our proposed dynamic keyframe extraction technique addresses these issues by intelligently identifying and extracting frames that represent significant behavioral changes, optimizing computational efficiency and accuracy in fatigue detection even in real-time or near-real-time applications.

#### 2.3.2 Mathematical principles

To deeply implement dynamic keyframe extraction, particularly considering rapidly changing lighting conditions, we have designed a series of complex mathematical models integrating calculus, optimization theory, and advanced statistical methods. Our goal is to accurately identify the driver’s fatigue state under various environmental conditions, especially in drastic lighting changes. Firstly, we define a multi-layer image sequence difference measure function *D*_*t*_ considering lighting changes:

$$\begin{array}{cc} D_{t} & =\;\frac{1}{2}\sum_{i=1}^{K}{\left\|\nabla^{2}\left(\sum_{p=1}^{P}\alpha_{p}\cdot (V_{t}^{p}-V_{t-i}^{p})\right)\right\|}^{2}\\ & \;+\lambda \sum_{q=1}^{Q}\beta_{q}\cdot (\sum_{r=1}^{R}\gamma_{r}\cdot {\left|\nabla (V_{t-q}^{r}-V_{t}^{r})\right|}^{3}) \end{array}$$ (8)

where $V_{t}^{p}$ and $V_{t-i}^{p}$ are the feature vectors of the image frames at times *t* and *t* − *i* at layer *p*, respectively, and *α*_*p*_, *β*_*q*_, *γ*_*r*_ are weight coefficients, with ∇^2^ and ∇ representing the second and first order derivatives, respectively. This multi-layer difference measurement helps in accurately capturing subtle changes in the image sequence caused by lighting variations, thus identifying keyframes with the most dynamic changes.

Secondly, we introduce a highly complex optimization model considering lighting changes for selecting the optimal set of keyframes *S*_*t*_:

$$\begin{array}{cc} L(S_{t},\theta ,\phi ,\psi ) & =\;\sum_{i=1}^{I}\theta_{i}\cdot (D_{t-i}-\phi \cdot \sum_{j=1}^{J}\kappa_{j}\cdot {\left\|\nabla (V_{t-j}-V_{t})\right\|}^{2})\\ & \;+\psi \cdot (\sum_{k=1}^{K}\omega_{k}\cdot (\sum_{l=1}^{L}\nu_{l}\cdot {\left|V_{t-k}^{l}-V_{t}^{l}\right|}^{4})-\delta ) \end{array}$$ (9)

where *θ*_*i*_, *ϕ*, *ψ*, *κ*_*j*_, *ω*_*k*_, *ν*_*l*_ are adjustment factors, and *δ* is a preset threshold. These adjustment factors allow the model to flexibly adapt during keyframe extraction, addressing various video sequences and environmental changes, including lighting variations.

Finally, we redefine our problem and propose a theorem about the effect of environmental factors, especially lighting changes, on fatigue prediction and the effectiveness of keyframe extraction.

**Problem 3**. *Given a series of time-series facial image frames, our task is to optimize a problem containing multi-layered, multi-dimensional feature difference measurements and a highly complex optimization model, to dynamically extract keyframes. This aims to accurately identify the driver’s fatigue state, especially in rapidly changing lighting conditions. The optimization problem can be reformulated as follows*,

$$\begin{array}{c} \underset{S_{t}}{\text{min}}\;\sum_{t=1}^{T}{\left(Y_{S_{t}}^{\prime }-{\hat{Y}}_{S_{t}}\right)}^{2}\\ subject\;to\;Y_{S_{t}}^{\prime }=\sigma (L\left(S_{t},\theta ,\phi ,\psi \right)+E_{S_{t}}) \end{array}$$ (10)

*where* $Y_{S_{t}}^{\prime }$ *is the fatigue state prediction output based on the selected keyframes*, $E_{S_{t}}$ *represents environmental factors, especially the impact of lighting changes on fatigue prediction, and* ${\hat{Y}}_{S_{t}}$ *is the actual fatigue state at time t*.

**Theorem 2** (Influence of Environmental Factors). *Environmental factors within the vehicle, such as lighting intensity L*_*t*_ *and noise level* *C*_*t*_, *significantly impact the accuracy of fatigue detection, which can be expressed by the following formula*:

$$\begin{array}{c} D_{t}\left(L_{t},C_{t}\right)=\frac{1}{2}\sum_{i=1}^{K}(\omega \left(L_{t},C_{t}\right)\cdot \sum_{p=1}^{P}\alpha_{p}\cdot {\left\|\nabla^{2}(V_{t}^{p}-V_{t-i}^{p})\right\|}^{2}\\ +\lambda \left(L_{t},C_{t}\right)\cdot \sum_{q=1}^{Q}\beta_{q}\cdot {\left|\nabla (V_{t-q}^{r}-V_{t}^{r})\right|}^{3}) \end{array}$$ (11)

*where* *ω*(*L*_*t*_, *C*_*t*_) *and* λ(*L*_*t*_, *C*_*t*_) *are functions of lighting intensity and noise level, respectively*.

**Lemma 1** (Effectiveness of Keyframe Extraction). *In the fatigue detection of video sequences, the dynamic keyframe extraction method is more effective than the static frame sampling method, mathematically expressed as*:

$$\begin{array}{c} L\left(S_{t},\theta ,\phi ,\psi ,L_{t},C_{t}\right)=\sum_{i=1}^{I}\theta_{i}\left(L_{t},C_{t}\right)\cdot (D_{t-i}\left(L_{t},C_{t}\right)\\ -\phi \left(L_{t},C_{t}\right)\cdot \sum_{j=1}^{J}\kappa_{j}\left(L_{t},C_{t}\right)\cdot {\left\|\nabla (V_{t-j}-V_{t})\right\|}^{2}) \end{array}$$ (12)

*where* *θ*_*i*_(*L*_*t*_, *C*_*t*_), *ϕ*(*L*_*t*_, *C*_*t*_), *κ*_*j*_(*L*_*t*_, *C*_*t*_) *are functions of the time window, lighting intensity, and noise level*.

Proof is provided in the S1 Appendix.

### 2.4 Composite action recognition network

#### 2.4.1 Motivation for composite action recognition network

- Traditional driver fatigue detection methods encounter limitations in capturing the nuanced spatiotemporal dynamics of continuous video frames, crucial for identifying subtle fatigue-related facial movements [22, 23]. Addressing these deficiencies, we introduce the Composite Action Recognition Network, merging the capabilities of 3D convolutional networks (3D CNN) and Long Short-Term Memory networks (LSTM). This integration is designed to process the spatial and temporal aspects of extracted keyframes dynamically, enhancing the accuracy and robustness of fatigue detection under variable lighting conditions.
- The use of 3D CNN in our approach might raise questions regarding its complexity and computational demand, particularly given the 3D model’s typical association with high-parameter requirements. However, it’s crucial to clarify that the “3D” aspect in our context refers to processing sequences of 2D images over time, extracted through the “Dynamic Keyframe Extraction” phase, rather than traditional 3-dimensional video data. This methodological choice enables us to efficiently capture the temporal dynamics and subtle changes in facial expressions with significantly reduced computational overhead, making it a practical solution for real-time fatigue detection applications.

#### 2.4.2 Mathematical principles

First, for the hybrid neural network architecture in the Composite Action Recognition Network, we constructed the following mathematical model.

**Hybrid Neural Network Architecture**:

$$\begin{array}{cc} H_{t} & =\;3DConv\left(V_{t},\Theta \right)+LSTM\left(V_{t},\Lambda \right)\\ & =\;\sum_{i=1}^{n}\theta_{i}\cdot \text{ReLU}(\sum_{j=1}^{J}W_{ij}*V_{t-j}+b_{i})\\ & \;+\sum_{k=1}^{m}\lambda_{k}\cdot (\sum_{l=1}^{L}U_{kl}\cdot V_{t-l}+c_{k}) \end{array}$$ (13)

$$\begin{array}{c} G_{t}=\;\sum_{i=1}^{p}\xi_{i}\cdot \text{Tanh}(\sum_{j=1}^{Q}Z_{ij}*H_{t-j}+d_{i}) \end{array}$$ (14)

$$\begin{array}{c} R_{t}=\;\sum_{i=1}^{r}\eta_{i}\cdot (\sum_{j=1}^{S}\text{Sigmoid}(Y_{ij}*G_{t-j}+e_{i})) \end{array}$$ (15)

where 3*DConv* and *LSTM* represent the 3D convolutional and Long Short-Term Memory layers, respectively, and Θ and Λ are their corresponding network parameter sets. Here, *θ*_*i*_, *W*_*ij*_, *b*_*i*_ are weights and biases of the 3D convolutional layer, while λ_*k*_, *U*_*kl*_, *c*_*k*_ are weights and biases of the LSTM layer. *G*_*t*_ integrates the outputs of both network layers through the Tanh activation function. *R*_*t*_ is the final output, using the Sigmoid function for the final classification result. This hybrid architecture design leverages the 3D convolutional layer’s capability to capture spatial features and the LSTM layer’s ability to process temporal sequence data.

**Additional Feature Extraction and Fusion Layers**:

$$\begin{array}{c} F_{t}=\;\sum_{i=1}^{s}\phi_{i}\cdot (\sum_{j=1}^{T}\text{ReLU}(A_{ij}*R_{t-j}+f_{i})) \end{array}$$ (16)

$$\begin{array}{c} C_{t}=\;\sum_{i=1}^{u}\psi_{i}\cdot (\sum_{j=1}^{V}\text{Sigmoid}(B_{ij}\cdot F_{t-j}+g_{i})) \end{array}$$ (17)

where *F*_*t*_ and *C*_*t*_ respectively represent additional feature extraction and fusion layers, further enhancing the network’s feature recognition capabilities. *ϕ*_*i*_, *A*_*ij*_, *f*_*i*_ are weights and biases of the feature extraction layer, while *ψ*_*i*_, *B*_*ij*_, *g*_*i*_ are weights and biases of the fusion layer.

Based on the above model, we define Problem 4:

**Problem 4**. *The goal of optimizing the hybrid neural network architecture is to maximize the spatiotemporal feature recognition capability of facial actions, while considering the complexity of dynamic keyframe extraction. The optimization problem can be expressed as*:

$$\begin{array}{cc} \underset{\Theta ,\Lambda ,\Xi ,}{\text{max}} & \;\sum_{t=1}^{T}(\alpha {\left(Y_{R_{t}}^{\prime \prime }-{\hat{Y}}_{t}\right)}^{2}+\beta (\sum_{s=1}^{S}{\left(M\left(F_{S_{t}},\Phi ,\Psi \right)-F_{S_{t}}^{\prime }\right)}^{2}))\\ subject\;to & \;Y_{R_{t}}^{\prime \prime }=\sigma (R_{t}+N\left(C_{t},\Xi ,\right))\\ & \;F_{S_{t}}^{\prime }=Tanh(L\left(S_{t},\theta ,\phi ,\psi \right)+E_{S_{t}}) \end{array}$$ (18)

*where* $Y_{R_{t}}^{\prime \prime }$ *is the fatigue state prediction output based on the hybrid neural network architecture*, ${\hat{Y}}_{t}$ *is the actual fatigue state at time t, and* $F_{S_{t}}^{\prime }$ *is the feature vector based on dynamic keyframe extraction*. *α*, *β* *are weighting factors used to balance the optimization of facial action recognition and keyframe extraction*.

*In this problem*, $L(S_{t},\theta ,\phi ,\psi )$ *represents the optimization model for dynamic keyframe extraction, integrating calculus, optimization theory, and advanced statistical methods*. $M(F_{S_{t}},\Phi ,\Psi )$ *and* $N(C_{t},\Xi ,)$ *respectively represent the complex mathematical models of additional feature extraction and fusion layers, further enhancing the model’s capability to process spatiotemporal data*. Θ, Λ, Ξ, Φ, Ψ *are sets of network parameters, covering weights and biases across all layers of the hybrid neural network architecture*.

#### 2.4.3 Fine-grained action recognition

Next, we explore fine-grained action recognition. **Fine-Grained Action Recognition:**

$$\begin{array}{cc} F_{t} & =\;\sum_{k=1}^{p}\omega_{k}\cdot \text{Sigmoid}(\sum_{l=1}^{L}Z_{kl}\cdot R_{t-l}+f_{k})\\ & =\;\sum_{k=1}^{p}\omega_{k}(\frac{1}{1+e^{-(\sum_{l=1}^{L}Z_{kl}\cdot R_{t-l}+f_{k})}}) \end{array}$$ (19)

$$\begin{array}{c} B_{t}=\;\sum_{k=1}^{q}\mu_{k}\cdot (\sum_{l=1}^{M}\text{Tanh}(N_{kl}\cdot F_{t-l}+g_{k})) \end{array}$$ (20)

$$\begin{array}{c} A_{t}=\;\sum_{k=1}^{r}\nu_{k}\cdot (\sum_{l=1}^{N}\text{ReLU}(O_{kl}\cdot B_{t-l}+h_{k})) \end{array}$$ (21)

$$\begin{array}{c} D_{t}=\;\sum_{k=1}^{s}\xi_{k}\cdot (\sum_{l=1}^{P}\text{LeakyReLU}(Q_{kl}\cdot A_{t-l}+i_{k})) \end{array}$$ (22)

where *ω*_*k*_, *Z*_*kl*_, *f*_*k*_, *μ*_*k*_, *N*_*kl*_, *g*_*k*_, *ν*_*k*_, *O*_*kl*_, *h*_*k*_, *ξ*_*k*_, *Q*_*kl*_, *i*_*k*_ are weights and biases of each layer. Here, Sigmoid, Tanh, and ReLU are activation functions, and LeakyReLU is an improved ReLU function, used to increase the network’s non-linearity and prevent the problem of gradient vanishing.

These equations collectively form the mathematical model for fine-grained action recognition. *F*_*t*_ represents the feature extraction of the first layer, using the Sigmoid function to process each neuron’s output. *B*_*t*_ is the second layer, using the Tanh activation function to extract more complex features. *A*_*t*_ is the third layer, employing the ReLU function to add non-linearity to the model. Finally, *D*_*t*_ uses the LeakyReLU function to further enhance the model’s expressive power. Each layer uses different activation functions and weighted sums to extract and merge features at different levels, thereby achieving precise recognition of subtle movements.

**Lemma 2** (Complexity of Feature Extraction). *Let a deep learning model* F *be used for feature extraction from video data. If* F *is sufficiently complex, it can more effectively extract fatigue-related features from video data. This can be described by the following mathematical expression*:

$$\begin{array}{c} F\left(V_{t}\right)=\sum_{i=1}^{s}\phi_{i}\cdot (\sum_{j=1}^{T}ReLU(A_{ij}*R_{t-j}+f_{i}))+\sum_{i=1}^{u}\psi_{i}\cdot (\sum_{j=1}^{V}Sigmoid(B_{ij}\cdot F_{t-j}+g_{i})) \end{array}$$ (23)

*where ϕ*_*i*_, *A*_*ij*_, *f*_*i*_ *are weights and biases of the additional feature extraction layer, and ψ*_*i*_, *B*_*ij*_, *g*_*i*_ *are weights and biases of the fusion layer. ReLU and Sigmoid are activation functions. This complex neural network structure can effectively extract complex features from time-series data*.

Proof is provided in the S1 Appendix.

Based on the mathematical model of fine-grained feature extraction, we define Problem 5:

**Problem 5**. *The goal of fine-grained action recognition is to distinguish subtle fatigue-related changes while capturing facial actions. The optimization problem can be represented as*:

$$\begin{array}{c} \underset{\Omega ,,,\Xi ,\Phi ,\Psi }{\text{max}}\;\sum_{t=1}^{T}(\gamma {\left(Y_{D_{t}}^{\prime \prime \prime }-{\hat{Y}}_{t}\right)}^{2}+\delta (\sum_{s=1}^{S}{\left(P\left(A_{S_{t}},\Xi ,\Phi \right)-A_{S_{t}}^{\prime }\right)}^{2})) \end{array}$$ (24)

$$\begin{array}{c} subject\;to\;Y_{D_{t}}^{\prime \prime \prime }=\sigma (D_{t}+Q\left(B_{t},,\Psi \right)) \end{array}$$ (25)

$$\begin{array}{c} \;A_{S_{t}}^{\prime }=LeakyReLU(L\left(S_{t},\Omega ,\right)+E_{S_{t}}) \end{array}$$ (26)

*where* $Y_{D_{t}}^{\prime \prime \prime }$ *is the fatigue state prediction output based on fine-grained feature extraction*, ${\hat{Y}}_{t}$ *is the actual fatigue state at time t, and* $A_{S_{t}}^{\prime }$ *is the feature vector based on dynamic keyframe extraction*. *γ*, *δ* *are weighting factors used to balance the optimization of fine-grained feature extraction and action recognition*.

*In this problem*, $L(S_{t},\Omega ,)$ *represents the optimization model for dynamic keyframe extraction, while* $P(A_{S_{t}},\Xi ,\Phi )$ *and* $Q(B_{t},,\Psi )$ *represent the complex mathematical models of additional feature extraction and fusion layers, enhancing the model’s capability to process spatiotemporal data*. Ω, Φ, Ψ *are sets of network parameters, covering weights and biases across all layers of the hybrid neural network architecture*.

Problem 5 focuses on fine-grained action recognition, aiming to precisely capture subtle facial changes of drivers, particularly those minor but critical signs of fatigue. This fine-grained recognition is crucial for improving the accuracy of fatigue driving detection. Our challenge is to adjust the network to sensitively respond to subtle facial movements, such as minor eye movements or brief gaze shifts. This not only requires complex mathematical models and optimization strategies but also a profound understanding of human behavior characteristics for effective and accurate detection of fatigue driving.

**Corollary 2** (Advantage of Composite Action Recognition). *Let* C *represent a 3D convolutional network*, L *a Long Short-Term Memory network, and* F *a Composite Action Recognition Network. In* F, *the 3D convolutional network* C *is responsible for capturing the spatial features of facial actions, while the Long Short-Term Memory network* L *handles time-series data, extracting temporal characteristics of movements. The Composite Action Recognition Network* F *combines the advantages of both, resulting in superior performance in recognizing fatigue signs in continuous video frames compared to using either network alone. This combination can be mathematically expressed as*:

$$\begin{array}{c} F\left(V_{t}\right)=\alpha \cdot 3DConv\left(V_{t},\Theta \right)+\beta \cdot LSTM\left(V_{t},\Lambda \right) \end{array}$$ (27)

*where* Θ *and* Λ *are the parameter sets of the 3D convolutional and Long Short-Term Memory networks, respectively, and α and β are coefficients for adjusting the importance of outputs from both networks. This combination enables* F *to capture instantaneous facial actions while also focusing on the evolution of movements over time, thereby enhancing the accuracy of fatigue sign recognition*.

Proof is provided in the S1 Appendix.

## 3 Algorithm pseudocode

### 3.1 Pseudocode and explanation for Algorithm 1

Algorithm 1 combines the concepts of Problem 1, Problem 2, and Problem 3.

**Algorithm 1**: Comprehensive Fatigue Detection Algorithm 1

 **Data**: Facial behavior video sequence

 **Result**: Preliminary fatigue detection feature vector set

**1 begin**

  // Content from **Problem 1**

**2**  Initialize the dynamic keyframe extraction model L, see Eqs (8) and (9)

  // Content from **Problem 2**

**3**  Initialize the composite action recognition network *H*_*t*_, *G*_*t*_, *R*_*t*_, see Eqs (13), (14) and (15)

  // Content from **Problem 3**

**4**   Initialize additional feature extraction and fusion layers *F*_*t*_, *C*_*t*_, see Eqs (16) and (19)

**5**   **for** *each time window* **do**

**6**    **foreach** *video frame* *V*_*t*_ **do**

**7**     Compute keyframe difference measure *D*_*t*_, see Eq (8)

**8**     **if** *keyframe extraction conditions are met* **then**

**9**       Extract keyframe set *S*_*t*_, based on optimization model L

**10**      Perform composite action recognition on each keyframe

**11**      **foreach** *keyframe* *S*_*t*_ **do**

**12**       Apply 3D convolution and LSTM networks, compute *H*_*t*_, see Eq (13)

**13**       Integrate feature extraction results, compute *G*_*t*_, see Eq (14)

**14**       Generate preliminary fatigue detection result, compute *R*_*t*_, see Eq (15)

**15**       Update additional feature extraction results, compute *F*_*t*_, see Eq (16)

**16**       Update fusion layer results, compute *C*_*t*_, see Eq (19)

**17**      **end**

**18**     **end**

**19**    **end**

**20**   **end**

**21**   **return** *Preliminary fatigue detection feature vector set*

**22 end**

### 3.2 Pseudocode and explanation for Algorithm 2

Algorithm 2 combines the concepts of Problem 4 and Problem 5, as well as the output of Algorithm 1.

**Algorithm 2**: Fine-Grained Fatigue Detection Algorithm 2

 **Data**: Preliminary fatigue detection feature vector set from **Algorithm 1**

 **Result**: Final fatigue detection result

**1** **begin**

   // Content from **Problem 4**

**2**   Initialize fine-grained action recognition models P,Q, see Eqs (25) and (26)

**3**   **foreach** *feature vector* *F*_*t*_ *from Algorithm 1* **do**

    // Content from **Problem 5**

**4**    Apply fine-grained action recognition model, compute *D*_*t*_, see Eq (22)

**5**    **for** *each time step t* **do**

**6**     Combine fine-grained features, update fatigue state prediction, see Eq (25)

**7**     Compute final fatigue detection result, see Eq (26)

**8**    **end**

**9**   **end**

**10**  **return** *Final fatigue detection result set*

**11** **end**

### 3.3 Time and space complexity of algorithms

#### 3.3.1 Algorithm 1

The time complexity of Algorithm 1 is mainly influenced by the complexity of keyframe extraction and the composite action recognition network. Assuming the length of the video sequence is *N*, the time complexity is *O*(*N* × *K* × *M*), where *K* is the number of keyframes in each time window, and *M* is the number of layers in the composite action recognition network. The space complexity is mainly determined by the storage of network parameters and intermediate feature vectors, estimated as *O*(*K* × *M*).

#### 3.3.2 Algorithm 2

The time complexity of Algorithm 2 is influenced by the fine-grained action recognition model. Assuming the size of the feature vector set output by Algorithm 1 is *P*, the time complexity is *O*(*P* × *L*), where *L* is the number of layers in the fine-grained action recognition model. The space complexity is mainly determined by the storage of model parameters and intermediate computation results, estimated as *O*(*L*).

## 4 Experiments

The proposed model is implemented using the PyTorch deep learning framework. All experiments are conducted on a workstation with a 2.10GHz Intel(R) Xeon(R) Silver 4116 CPU, 16GB RAM, an NVIDIA Tesla V100 GPU, and Ubuntu 16.04.

To evaluate the performance of the fatigue detection system, a series of tests were conducted under various experimental conditions. Experimental parameters include the characteristics of the dataset, model training parameters, keyframe extraction settings, etc. Table 2 details the experimental parameter settings. Data augmentation techniques, including rotation and flipping, significantly improve our model’s generalization capability. By introducing variations in facial orientation and posture, these techniques ensure robustness against the diverse manifestations of driver fatigue. This diversity in the training data helps prevent overfitting, enabling the model to recognize fatigue-related features across different individuals and scenarios effectively, thus enhancing detection efficiency.

**Table 2:** Experimental parameter settings for the fatigue detection system.

Parameter	Value
Dataset Size	1000 video clips
Video Resolution	1920x1080 pixels
Average Duration per Video	10 minutes
Time Window Size *N*	30 seconds
Frame Extraction Frequency	5 15 frames/sec
Data Augmentation	Rotation, Flipping
Positive Sample Ratio	60%
Negative Sample Ratio	40%
CNN Layers	4 layers
LSTM Layers	2 layers
Filters per Layer	64
Activation Function	ReLU
Feature Extraction Layer Parameters Φ	256 neurons
Fusion Layer Parameters Ψ	128 neurons
Light Intensity Adjustment Factor *α*	0.5
Vehicle Noise Adjustment Factor *β*	0.3
Training Epochs	150 epochs
Batch Size	32
Learning Rate	0.001
Optimizer	Adam
Loss Function	Cross-Entropy Loss
Dropout Rate	0.5
Validation Set Proportion	20%
Early Stopping Threshold	10 epochs
Regularization Method	L2 Regularization
L2 Regularization Rate	0.01
Data Augmentation Techniques	Random Rotation, Scaling
Rotation Range	±15 degrees
Scaling Range	0.8–1.2
Input Data Format	Color Images
Image Channels	3 (RGB)
Keyframe Extraction Strategy	Content-Based
Evaluation Metrics	Accuracy, Recall
Test Set Proportion	20%
Data Preprocessing	Normalization, Denoising
Denoising Method	Gaussian Filtering
Normalization Range	0–1
Experimental Environment	NVIDIA Tesla V100 GPU

### 4.1 Datasets

#### 4.1.1 Yawn dataset

The Yawn Dataset, available on Kaggle’s official website, contains two categories: Yawn and no-Yawn. The Yawn category includes 2528 JPG format images, while the no-Yawn category includes 2591 JPG format images. Each type features “mouth” characteristics from different races, genders, and ages. The data is split into a training set and a test set in a 4:1 ratio to train and validate the two algorithms proposed in this paper.

#### 4.1.2 YawDDR dataset

The YawDDR dataset is an extension of the standard YawDD dataset, which is a publicly available dataset for yawning detection. The YawDDR dataset serves as a benchmark for evaluating face detection, feature extraction, and yawning detection algorithms. This dataset is composed of 351 video clips featuring a variety of volunteers who differ in gender, age, nationality, and ethnicity. Captured within the confines of stationary vehicles under daylight, these videos exhibit subtle differences in lighting conditions. The dataset records each participant in three to four separate videos, showcasing a range of oral movements including talking, yawning, and a combination of both. Given the variety of facial expressions and the need for clarity in data analysis, these videos, often longer than a minute and containing numerous facial movements, have been divided into smaller segments, thus forming the comprehensive YawDDR dataset.

**Fig 1:**
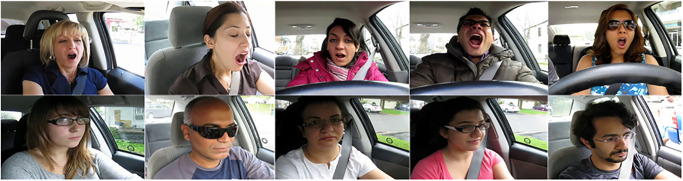
Example of YawDDR dataset (Image source: Yawning detection dataset (DOI: https://dx.doi.org/10.21227/e1qm-hb90), licensed under CC BY 4.0.).

**Fig 2:**
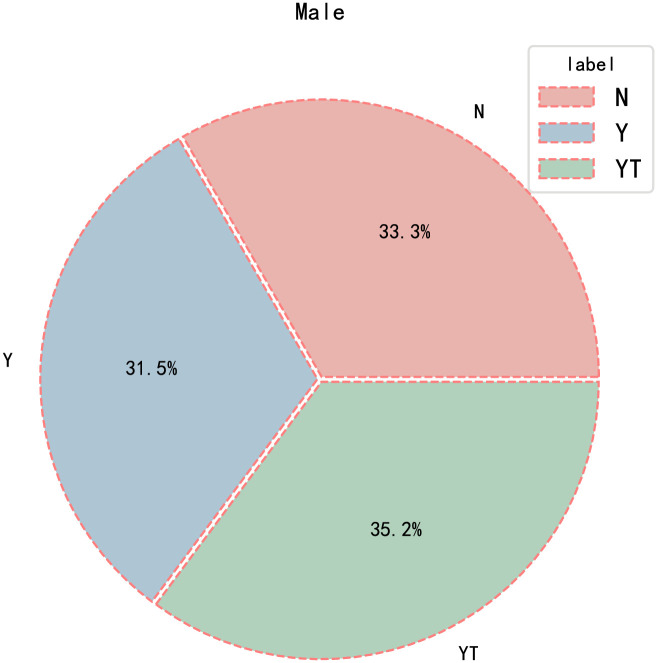
Label statistical results of YawDDR dataset. (N: Normal no yawning; Y: Yawning; YT: Yawning & Talking).

The video length in the YawDDR dataset is approximately 8 seconds, covering three distinct actions: talking, yawning, and yawning while talking. Sample images from the dataset before face segmentation are shown in Fig 1. Fig 2 presents 486 image sequences from the YawDDR dataset. The YawDDR dataset is used to validate the efficacy of the proposed method.

### 4.2 Experimental design and results

#### 4.2.1 Model performance testing

**Fig 3:**
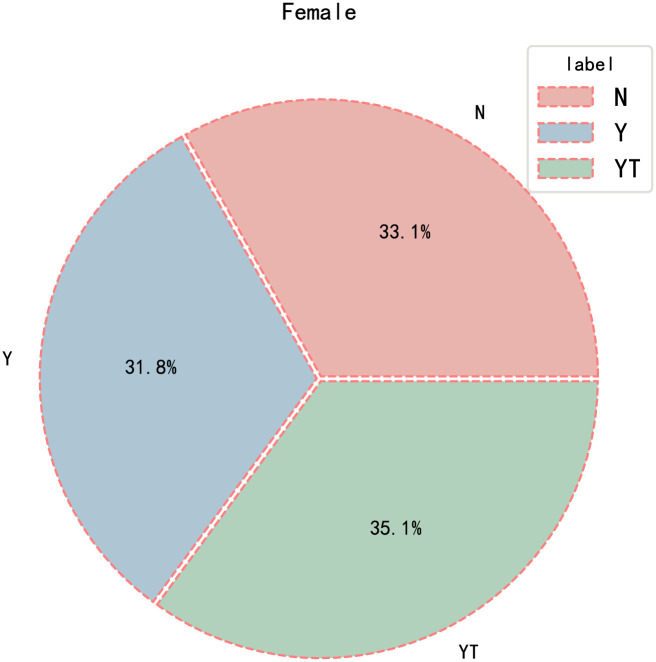
Nomal examples (Image source: Yawning detection dataset (DOI: https://dx.doi.org/10.21227/e1qm-hb90), licensed under CC BY 4.0.).

**Fig 4:**
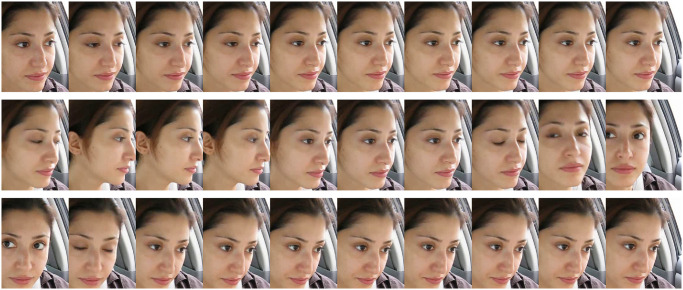
Talking examples (Image source: Yawning detection dataset (DOI: https://dx.doi.org/10.21227/e1qm-hb90), licensed under CC BY 4.0.).

**Fig 5:**
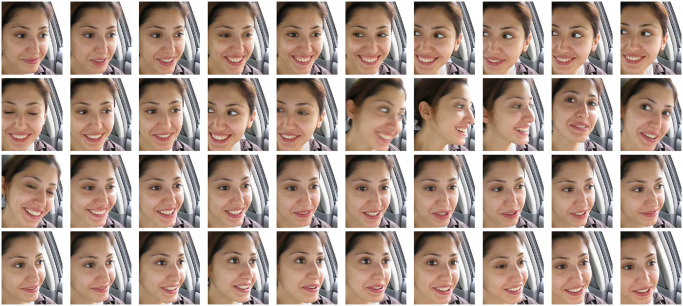
Yawning examples (Image source: Yawning detection dataset (DOI: https://dx.doi.org/10.21227/e1qm-hb90), licensed under CC BY 4.0.).

To test the two algorithms proposed in this paper, the YawDDR dataset was first processed using dynamic keyframe extraction. This method reduced the original 8-second videos with over 100 frames to data groups of about 30 frames each, ensuring no duplicate images in each group. Subsequently, facial information was extracted from each keyframe. Figs 3–5 show the keyframe cropping results for the YawDDR dataset, where a represents keyframes under Normal state, Talking state, and Yawning state.

#### 4.2.2 Impact of brightness on the two Algorithms

**Fig 6:**
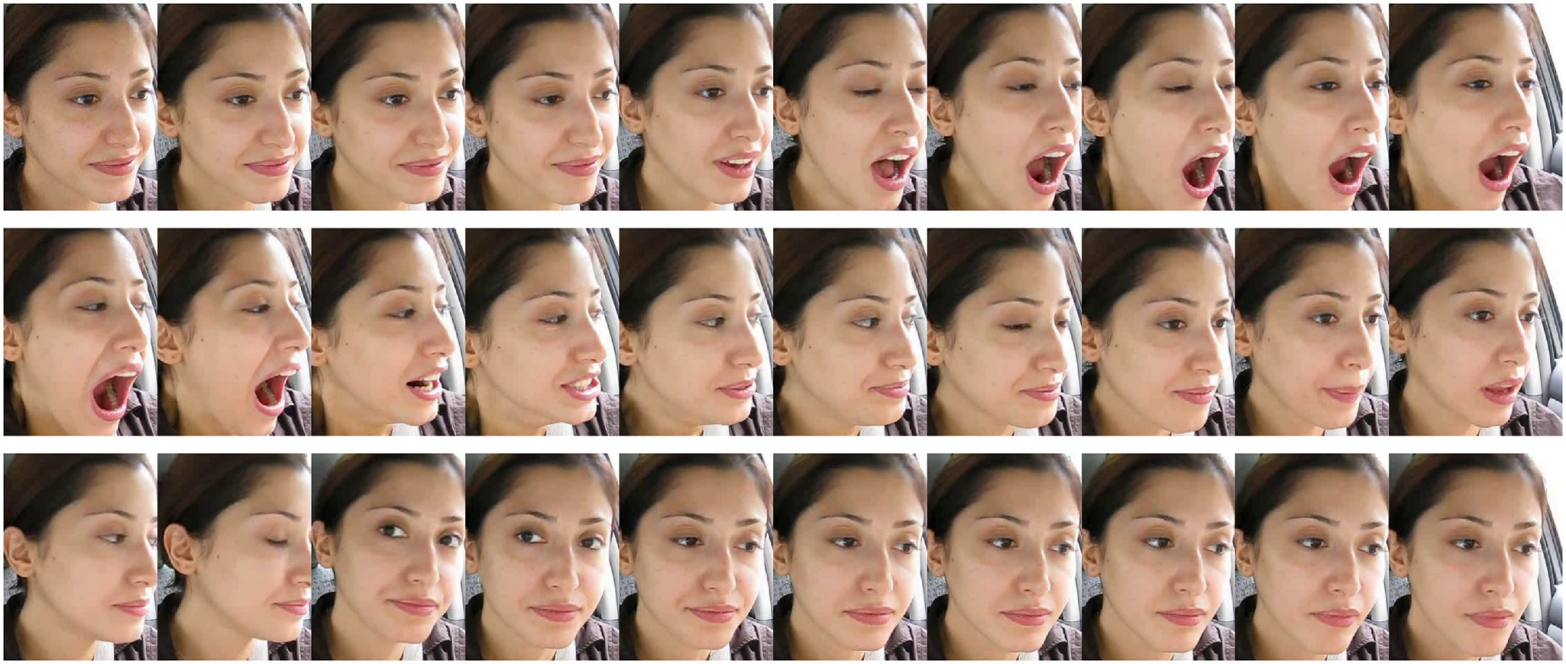
Label statistical results of YAWDDR dataset (Part 1/3, (Image source: Yawning detection dataset (DOI: https://dx.doi.org/10.21227/e1qm-hb90), licensed under CC BY 4.0.)).

**Fig 7:**
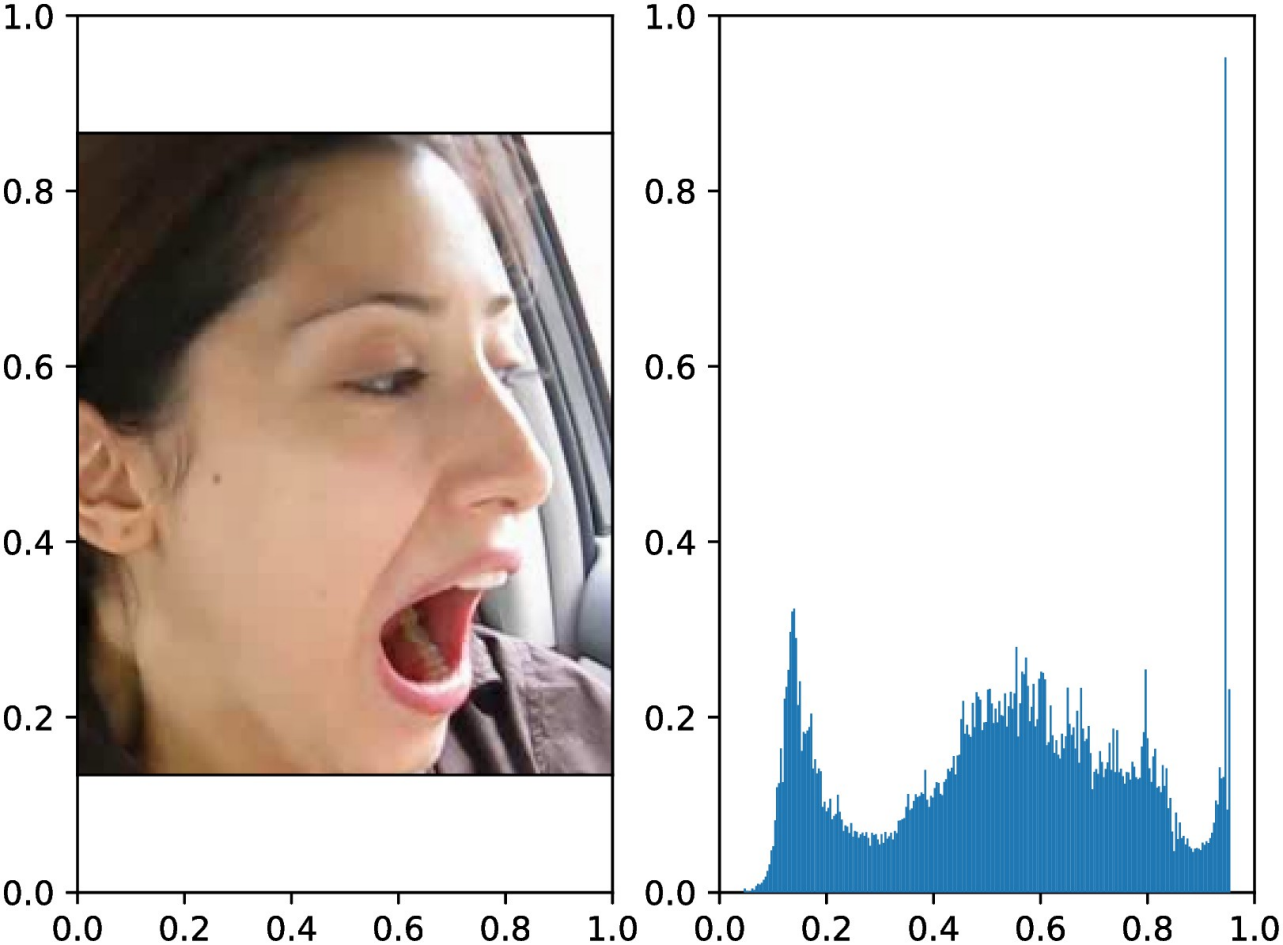
Label statistical results of YAWDDR dataset (Part 2/3, (Image source: Yawning detection dataset (DOI: https://dx.doi.org/10.21227/e1qm-hb90), licensed under CC BY 4.0.)).

**Fig 8:**
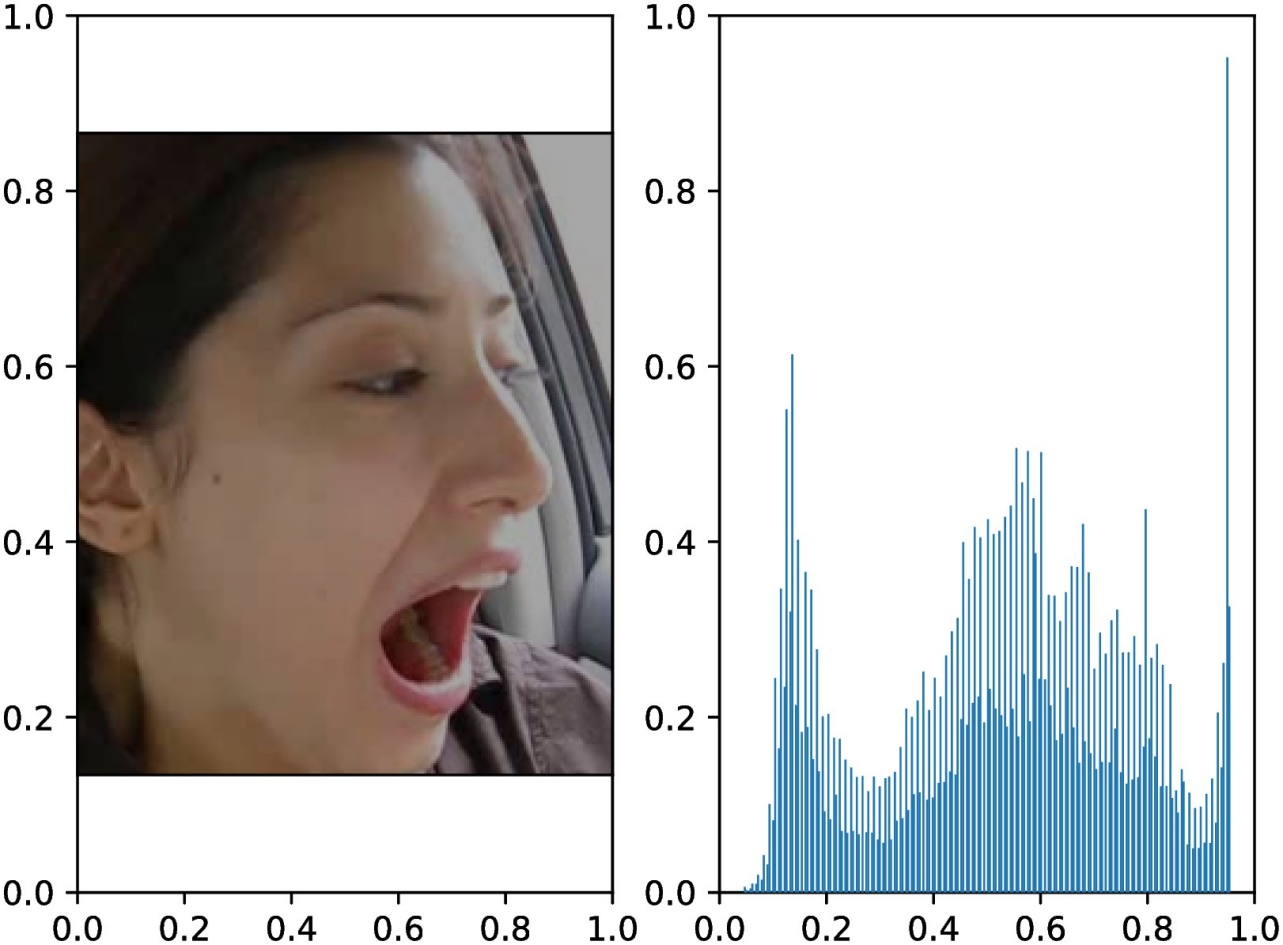
Label statistical results of YAWDDR dataset (Part 3/3, (Image source: Yawning detection dataset (DOI: https://dx.doi.org/10.21227/e1qm-hb90), licensed under CC BY 4.0.)).

To validate the point proposed in this paper, the brightness of each keyframe in the YawDDR dataset was processed. Figs 6–8 show examples of the YawDDR dataset after brightness processing. (a) is an image with 100% brightness, (b) is an image with 70% brightness, and (c) is an image with 130% brightness.

**Fig 9:**
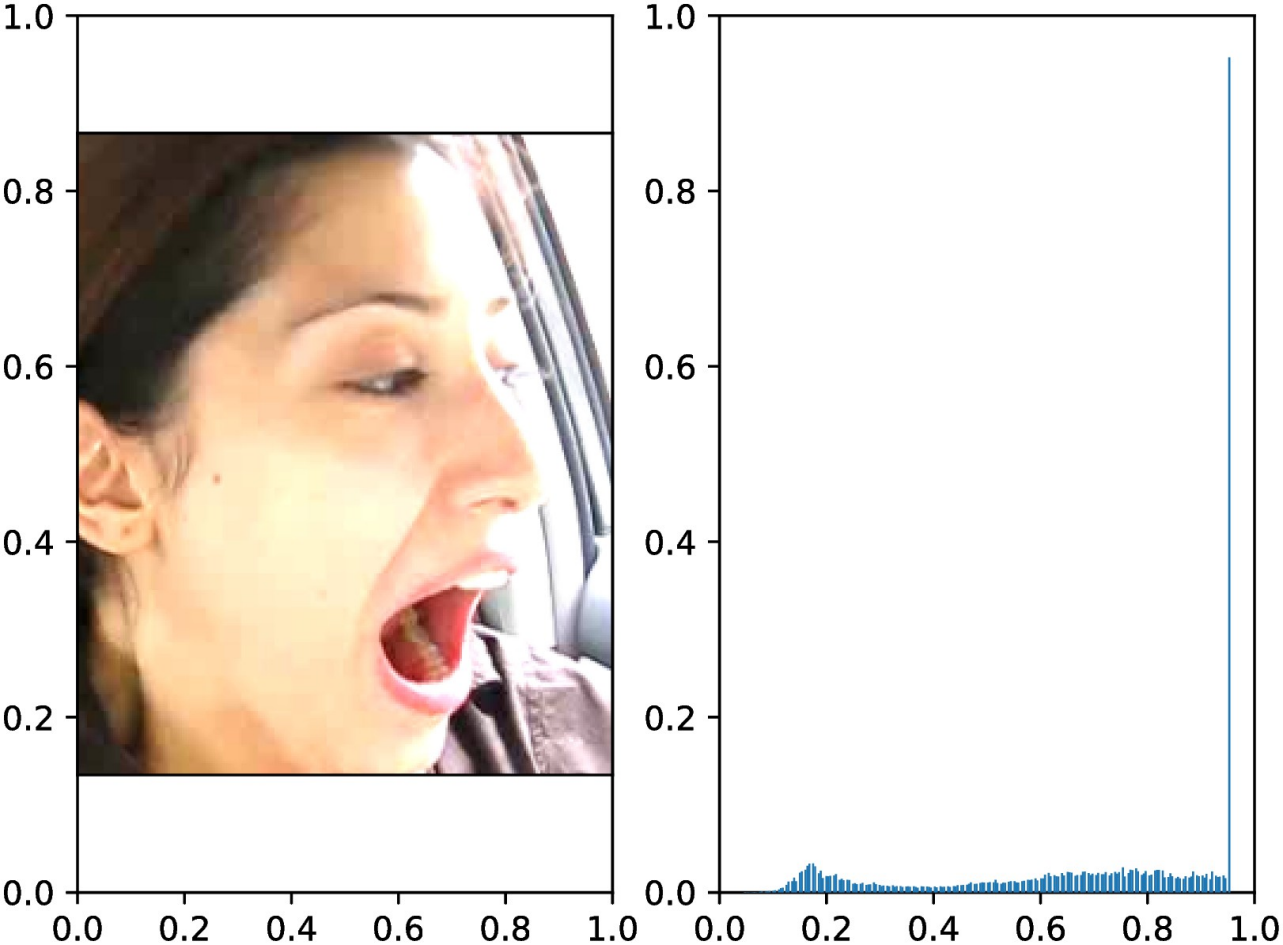
Accuracy of Algorithm1 and Algorithm2.

**Fig 10:**
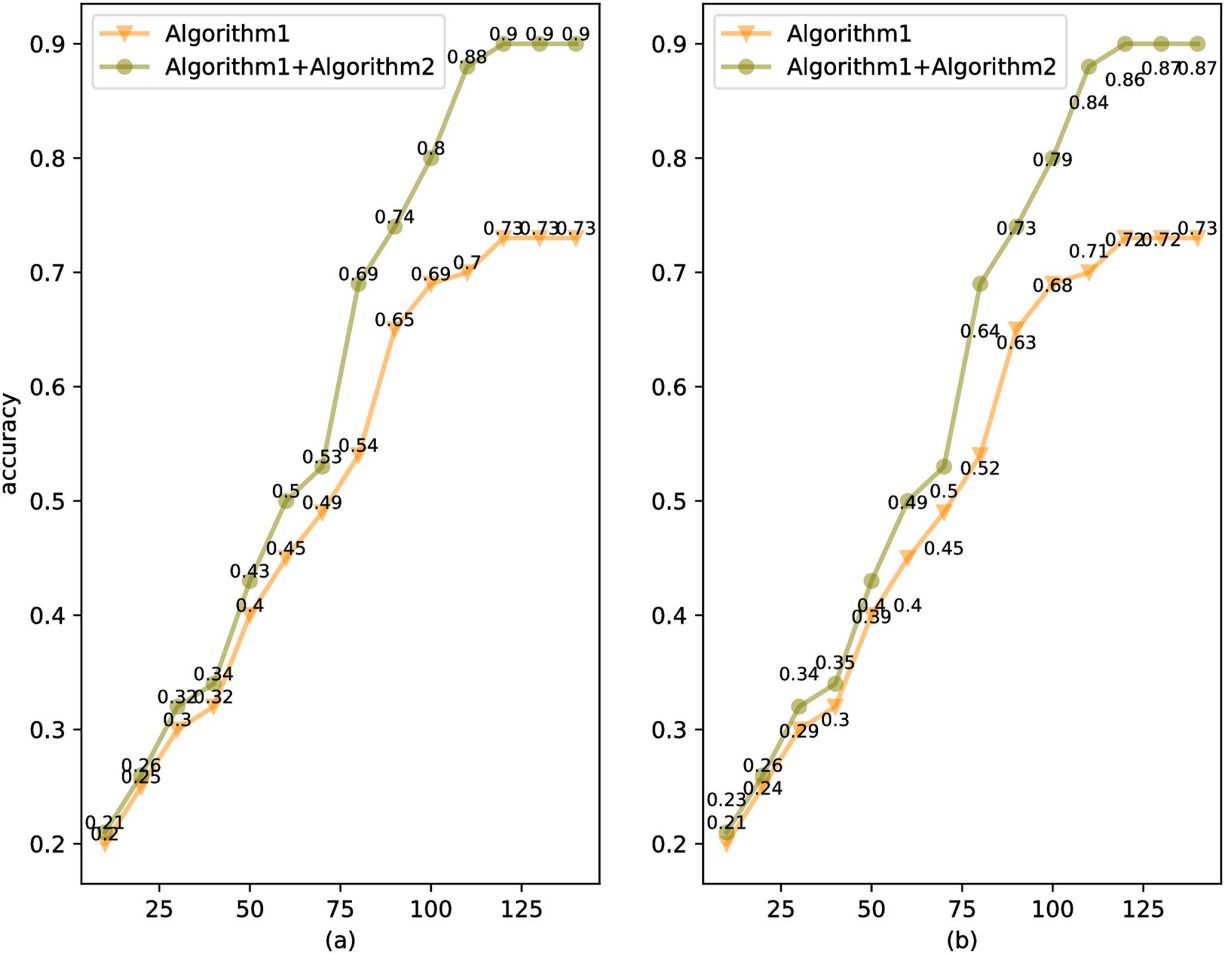
Experimental precision, recall, and F1 score results.

Models were trained and tested on the Yawn and YawDDR datasets, respectively. Fig 9 shows the detection accuracy of Algorithms 1 and 2 on the Yawn and YawDDR datasets in the test set, meanwhile, Fig 10 shows the detected confusion matrix results of Algorithms 1 and 2 on the Yawn and YawDDR datasets in the test set, The experimental results indicate that Algorithm 2, proposed in this paper, has a higher detection precision than Algorithm 1, demonstrating that the improvements made in Algorithm 2 have a positive effect. Both algorithms performed better on the Yawn dataset compared to the YawDDR dataset. This is attributed to the Yawn dataset containing only mouth features, which provide less information and make classification detection easier. Table 3 presents a comparison of accuracy between this paper and other studies.

**Table 3:** Comparison of Algorithm accuracy.

Method	Accuracy
Our Method	95.3
Our Method without Algorithm 2	90.1
Kielty et al. (2023) [24]	92.0
Yang et al. (2020) [25]	83.4
Majeed et al. (2023) [26]	95.0
Mzoughi et al. (2020) [27]	89.8
Kalfaoglu et al. (2020) [28]	90.8
Zhao et al. (2021) [29]	81.9

**Fig 11:**
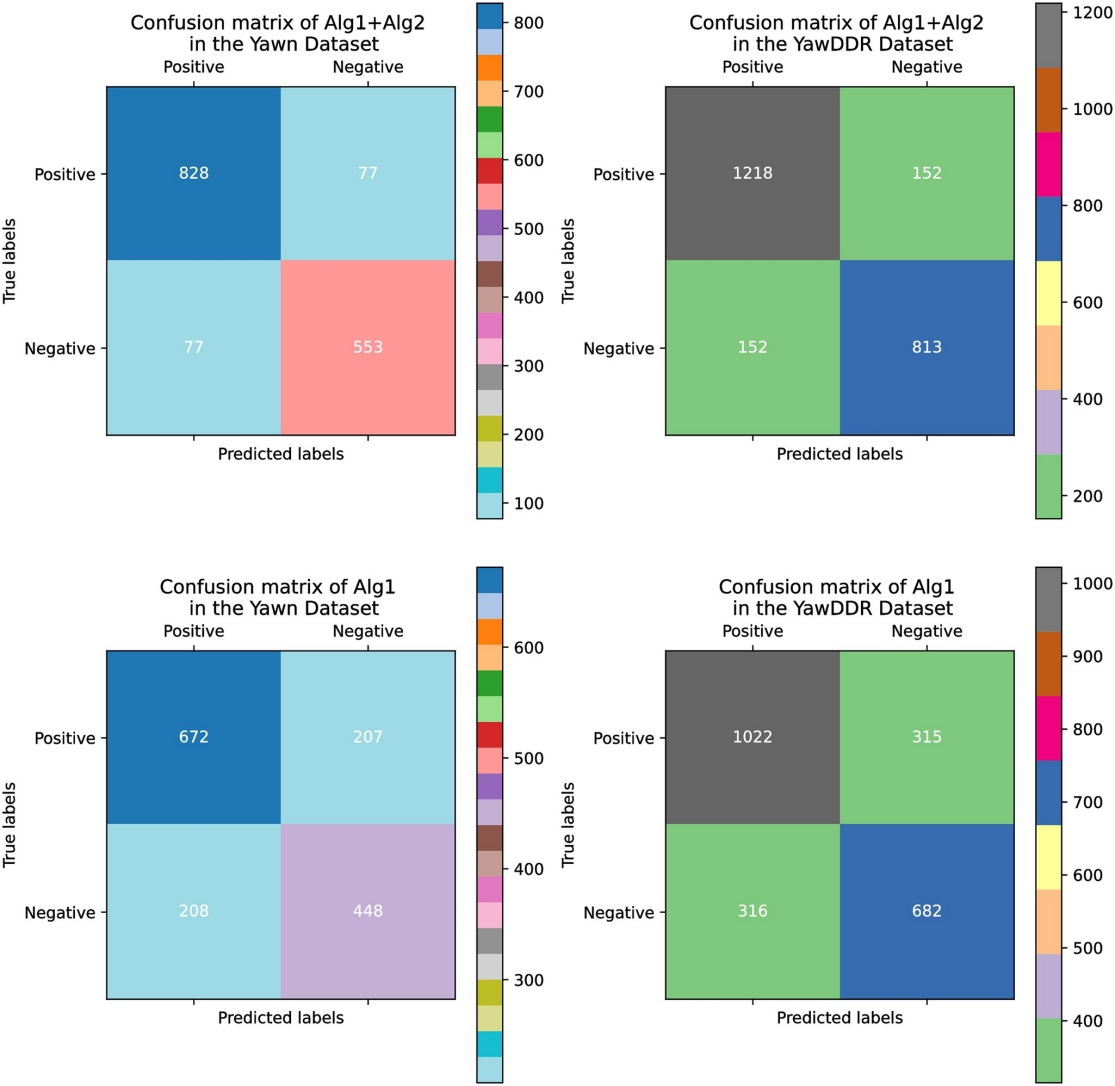
Confusion matrix results.

Then we analyzed the precision, recall and F1 score of the experimental results as shown in Fig 11. For these two data sets, Algorithm 2 is always better than Algorithm 1 in all three indicators (precision, recall and F1 score). In terms of accuracy, Algorithm 2 reaches peaks, especially on the Yawn dataset, demonstrating its increased ability to correctly identify positive instances. The recall metric measures the algorithm’s ability to find all actual positive instances and also shows the superior performance of Algorithm 2. Algorithm 2 is shown to be more efficient at capturing all relevant instances and does not lose as many true positives as Algorithm 1. The F1 score is a balanced metric that combines precision and recall, and Algorithm 2 scores significantly higher on both datasets. It is shown that Algorithm 2 has a better overall balance between precision and recall, indicating that it is a more robust algorithm in terms of correctness and completeness in handling detection tasks. In short, it shows that our algorithm can show the superiority of our algorithm no matter which index is used.

#### 4.2.3 Impact of brightness changes on Algorithm 2

Table 4 presents the detection results of the two models proposed in this paper on the YAWDDR dataset. The data indicates that the brightness of images affects the model’s detection results. In other words, in a visible environment, lower interior brightness leads to lower detection results. However, with sufficient interior light, the model’s detection accuracy improves by 0.01%-0.05%.

**Table 4:** Detection accuracy between the proposed method on YAWDDR dataset. (Ave: Average; Y: Yawning; YT: Yawn & Talking; T: Talking).

Brightness Level	Method	Ave	Y	YT	T
70%	Algorithm 1	90.5	91.2	90.2	90.3
	Algorithm 1+Algorithm 2	91.2	91.3	90.2	91.4
100%	Algorithm 1	91.3	91.3	92.0	90.1
	Algorithm 1+Algorithm 2	95.3	94.7	96.3	95.1
130%	Algorithm 1	92.2	91.6	90.4	92.0
	Algorithm 1+Algorithm 2	95.6	95.1	95.4	96.2

To further observe the impact of interior lighting changes on the accuracy of the model, three levels of brightness were set: 70% as low brightness, 100% as medium brightness, and 130% as high brightness. To simulate changes in vehicle lighting, the keyframes were further processed to set varying levels of brightness, namely strong-medium-weak (HML), strong-weak-medium (HLM), medium-strong-weak (MHL), medium-weak-strong (MLH), weak-strong-medium (LHM), and weak-medium-strong (LMH). Figs 6–8 illustrate the keyframe changes in the YawDDR dataset with weak-medium-strong brightness.

**Fig 12:**
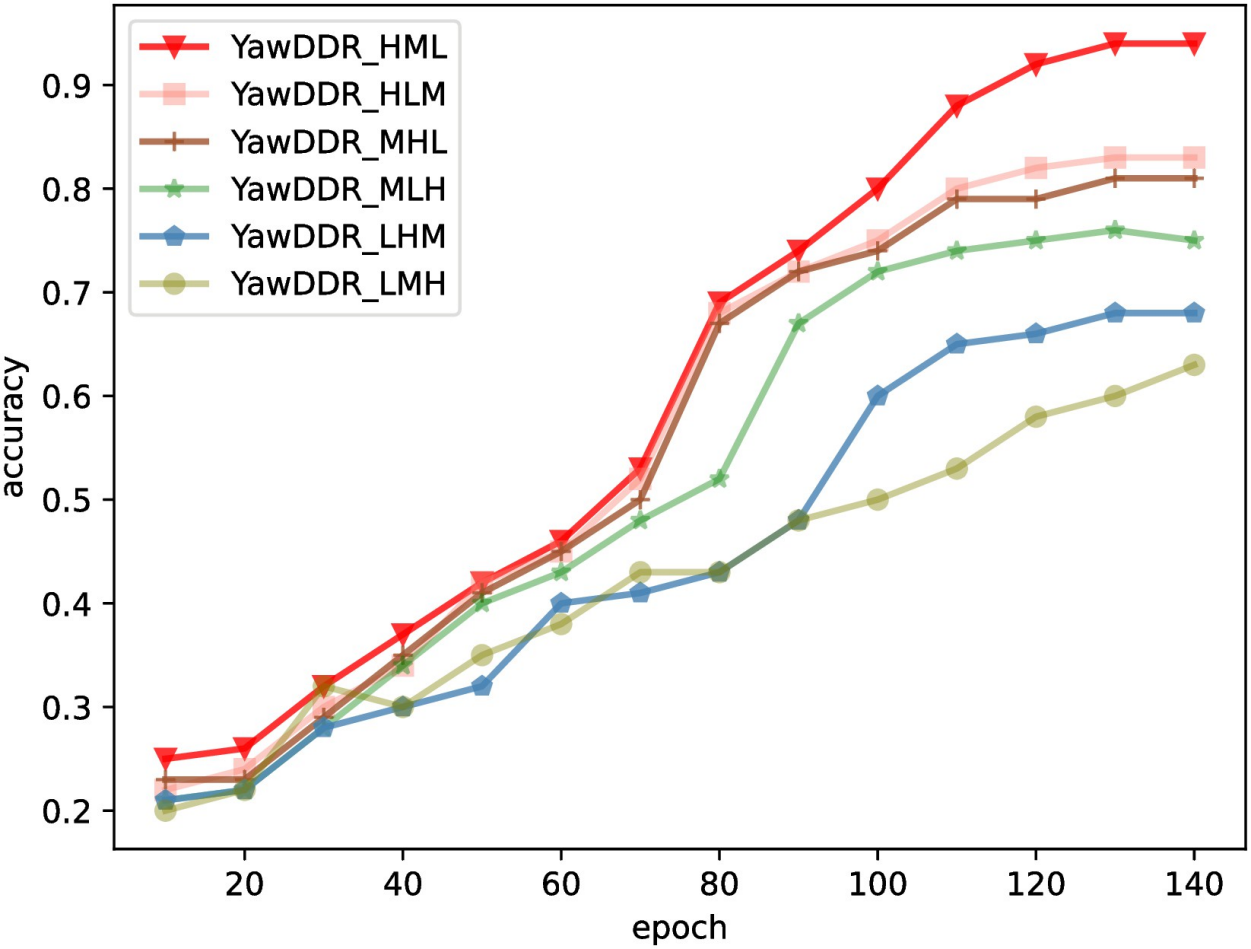
The accuracy of Algorithm1 and Algorithm2.

Using the above method, datasets were processed as YawDDR_HML, YawDDR_HLM, YawDDR_MHL, YawDDR_MLH, YawDDR_LHM, and YawDDR_LMH. Algorithm 2 was trained using the same method, and the model detection results are shown in Fig 12. The graph indicates that the overall accuracy of the algorithm decreases when brightness variation is introduced. When the lighting change pattern is HML, the model experiences less interference and achieves higher accuracy. Inferring from the results of the second experiment, the strong light at the beginning aids model detection, while the weak light in the latter half has a minor impact. Consequently, HLM and HLM brightness variations yield higher detection accuracy than MHL and MLH, which in turn are more accurate than LHM and LMH.

### 4.3 Discussion

- The generalization of our model to real-time datasets presents a significant challenge, primarily due to the inherent variability in such environments, including fluctuating lighting conditions, diverse driver behaviors, and unpredictable external factors. While our current study demonstrates promising results on the Yawn and YawDDR datasets, real-world application scenarios might introduce complexities not fully captured by these datasets. The limitations of our study in handling rapidly changing lighting conditions in a real-car environment highlight the need for further research. Future work could explore the integration of adaptive algorithms capable of dynamically adjusting to varying environmental conditions to improve real-time applicability.
- Assessing driving fatigue under “challenge” conditions goes beyond lighting variations. Factors such as the driver’s face distance from the camera, orientation, and possible occlusions (e.g., sunglasses or other facial wear) can significantly affect the classification accuracy. Our current methodology does not explicitly account for these variables, which may impact the model’s performance in real-world scenarios. Acknowledging these limitations is crucial for guiding future enhancements of our fatigue detection system. Efforts to include more diverse and challenging conditions in our training and validation datasets will be vital for improving the robustness and reliability of the model.
- Looking ahead, potential developments for our work include exploring multi-modal data integration, such as combining visual cues with physiological signals (e.g., heart rate or skin conductance) for a more comprehensive assessment of driver fatigue. Additionally, implementing our proposed method in real-car testing under various operational conditions will be essential to evaluate its effectiveness in real-world scenarios. This practical assessment will help identify specific areas for improvement, particularly in dealing with rapidly changing lighting conditions and other environmental factors not fully simulated in controlled datasets.

## 5 Conclusion

This study proposes an innovative method for driver fatigue detection, aiming to enhance the safety and reliability of intelligent driving assistance systems. By combining CNN model with emotional state analysis, our approach demonstrates outstanding robustness in complex environments with varying illumination. The experimental results show that our method significantly improves both accuracy and computational efficiency compared to existing technologies, particularly in environments with drastic changes in lighting conditions. Furthermore, this study explores the impact of different lighting conditions on fatigue detection accuracy, finding that changes in brightness significantly affect model performance. Especially in experiments simulating interior lighting changes, the results indicate that different combinations of light intensity have varying effects on model accuracy, providing crucial insights for optimizing fatigue detection models in such environments.

Overall, this study not only proposes an effective method for driver fatigue detection but also provides robust evidence for the adaptability and reliability of intelligent driving assistance systems in complex environments. We anticipate that this approach can be further optimized and applied to make greater contributions to road safety and driver welfare.

## Supporting information

S1 Appendix (PDF)
