# Supplementary material for: Advancing driver fatigue detection in diverse lighting conditions for assisted driving vehicles with enhanced facial recognition technologies
Source: PLoS One. 2024 Jul 10;19(7):e0304669. doi: 10.1371/journal.pone.0304669 (PMC11236172; doi:10.1371/journal.pone.0304669)
Supplement: S1 Appendix — (PDF) [file pone.0304669.s001.pdf]

Appendix: Mathematical Theorems, Reasoning, Lemmas, and Corollaries with Proofs

Theorem 1 (Accuracy of Fatigue Detection). *For any given continuous video sequence, if there exists a time window size N and a sufficiently complex feature extraction network, the driver's fatigue state can be accurately identified through this network. Specifically, if the feature extraction network can maximize the joint information entropy $H(V_t, E_t)$ between facial feature vectors V_t and emotional state E_t, the recognition of fatigue state will be more accurate.*

Proof. Assume the opposite, that is, for a given continuous video sequence and time window size N, even with a sufficiently complex feature extraction network, it is not possible to accurately identify the driver's fatigue state. Let $\mathcal{F}$ be the feature extraction network that operates on the video frame sequence $V = \{v_1, v_2, \ldots, v_N\}$ within the time window and outputs fatigue state estimates $Y = \{y_1, y_2, \ldots, y_N\}$.

Considering that $\mathcal{F}$ is a complex feature extraction network, it should be able to identify subtle variations related to the fatigue state. If $\mathcal{F}$ cannot distinguish the fatigue state, there exist consecutive frames v_t and v_{t+1} such that:

$$\|\mathcal{F}(v_t) - \mathcal{F}(v_{t+1})\|_2^2 < \epsilon \tag{1}$$

where ϵ is a small positive value.

However, for significant fatigue state changes, $\mathcal{F}$ should produce significantly different outputs. This implies that for the joint information entropy $H(V_t, E_t)$ of V_t and E_t, there should be a threshold Θ:

$$H(V_t, E_t) > \Theta \tag{2}$$

Since the design purpose of $\mathcal{F}$ is to capture subtle variations in the fatigue state, it should be able to produce significantly different outputs for significant fatigue variations, leading to a significant increase in joint information entropy. This implies:

$$\|\mathcal{F}(v_t) - \mathcal{F}(v_{t+1})\|_2^2 > \lambda \cdot H(V_t, E_t) \tag{3}$$

where λ is a positive coefficient representing the amplification capability of $\mathcal{F}$ for fatigue feature variations.

Combining equation (1) and equation (3), we obtain a contradiction:

$$\epsilon > \lambda \cdot \Theta \tag{4}$$

which contradicts the significant fatigue state change $H(V_t, E_t) > \Theta$. Therefore, our original assumption is incorrect. Thus, for any given continuous video sequence, if there exists a time window size N and a sufficiently complex feature extraction network $\mathcal{F}$, the driver's fatigue state can be accurately identified through this network.

Theorem 2 (Influence of Environmental Factors). *Environmental factors within the vehicle, such as light intensity L_t and noise level C_t, have a significant impact on fatigue detection accuracy, which can be expressed by the following formula:*

$$\begin{aligned} D_t(L_t, C_t) = \frac{1}{2} \sum_{i=1}^{K} \Bigg(& \omega(L_t, C_t) \cdot \sum_{p=1}^{P} \alpha_p \cdot \left\| \nabla^2 \left(V_t^p - V_{t-i}^p \right) \right\|^2 \\ & + \lambda(L_t, C_t) \cdot \sum_{q=1}^{Q} \beta_q \cdot \left| \nabla (V_{t-q}^r - V_t^r) \right|^3 \Bigg) \end{aligned} \tag{5}$$

where $\omega(L_t, C_t)$ and $\lambda(L_t, C_t)$ are functions of light intensity and noise level.

Proof. To prove this theorem, we consider the complex dynamic response of the fatigue detection system when dealing with environmental factors within the vehicle, such as light intensity L_t and noise level C_t. We introduce a multivariate function F that estimates the fatigue state Y_t by combining the driver's facial features V_t, physiological signals S_t, and environmental factors L_t and C_t.

Define the fatigue detection state Y_t as follows:

$$Y_t = F(V_t, S_t, L_t, C_t) \tag{6}$$

where F represents the multi-input multi-output function of fatigue detection.

We further define the comprehensive impact function $E(L_t, C_t)$, which translates light intensity and noise level into an environmental impact measure:

$$E_t = E(L_t, C_t) = \omega(L_t, C_t) \cdot D_t(L_t, C_t) \tag{7}$$

where $D_t(L_t, C_t)$ is as defined in the theorem.

Considering the dynamics of F over a continuous time window, we define the change in environmental impact ΔE:

$$\Delta E = E_{t+1} - E_t \tag{8}$$

Assuming that F can accurately capture the dynamic changes in environmental factors, then for significant environmental changes ΔE, we have:

$$\|F(V_{t+1}, S_{t+1}, L_{t+1}, C_{t+1}) - F(V_t, S_t, L_t, C_t)\|_2^2 > \alpha \cdot \|\Delta E\|_2^2 \tag{9}$$

where α is a positive coefficient representing the sensitivity of F to environmental changes.

Therefore, by demonstrating that F can capture significant fatigue state changes in its output due to environmental changes ΔE, we prove that environmental factors within the vehicle, such as light intensity L_t and noise level C_t, have a significant impact on fatigue detection accuracy.

Lemma 1 (Effectiveness of Key Frame Extraction)**.** *In fatigue detection of video sequences, dynamic key frame extraction methods are more effective than static frame sampling methods, and their mathematical expression is as follows:*

$$KF_t = \arg\max_i \left(H(V_i, E_i) - \beta \cdot \sum_{j=1}^{N} \|V_i - V_{i-j}\|_2^2 \right) \tag{10}$$

where KF_t represents the selected key frame at time t, $H(V_i, E_i)$ represents the joint information entropy, β is a positive coefficient, and N is the maximum time delay allowed.

Corollary 1 (Improved Fatigue Detection Accuracy)**.** *Utilizing the key frame extraction method defined in Lemma 3 results in improved fatigue detection accuracy compared to static frame sampling methods.*

Proof. The proof of this corollary follows from Lemma 3. By selecting key frames based on the proposed method, we prioritize frames with high joint information entropy $H(V_i, E_i)$ while considering the temporal relationship between frames. This approach captures the most informative frames related to the driver's fatigue state.

Since these key frames are selected with a focus on capturing the driver's fatigue-related features, the accuracy of fatigue detection is improved compared to static frame sampling methods, which do not consider such temporal relationships. Thus, the corollary is proven.
